# First observation of larval oarfish, *Regalecus russelii*, from fertilized eggs through hatching, following artificial insemination in captivity

**DOI:** 10.1186/s40851-020-00156-6

**Published:** 2020-04-08

**Authors:** Shin-ichiro Oka, Masaru Nakamura, Ryo Nozu, Kei Miyamoto

**Affiliations:** Okinawa Churashima Foundation, 888 Ishikawa, Motobu-cho, Okinawa, 905-0206 Japan

**Keywords:** *Regalecus*, Artificial fertilization, Development, Deep-sea fish

## Abstract

**Background:**

Little is known about the life history of oarfish of the genus *Regalecus*, although it is a famous deep-sea fish and an apparent origin of sea serpent legends. We successfully performed artificial insemination using a recently dead pair of sexually mature individuals. We report for the first time development from fertilized eggs to early larvae in the Lampridiformes.

**Results:**

Eggs required 18 days of development from fertilization to hatching under 20.5–22.5 °C conditions. Oarfish larvae had similar morphological features as other lampridiform larvae hatched in the ocean. Larvae typically faced downward and swam using pectoral fins; they frequently opened their mouths. This mouth-opening behavior and swimming ability were both consistent with osteological development. The larvae did not eat and died four days after hatching.

**Conclusions:**

This is the first successful instance of artificial insemination and hatching in the oarfish, as well as the first reliable morphological and behavioral description of lampridiform larvae.

## Background

The oarfish of the genus *Regalecus* is found in deep seas from tropical to temperate zones and is usually mesopelagic [1]. *Regalecus russelii* has a unique morphology and a large body; much of the information about it comes from findings of dead bodies on beaches [2–4]. Beyond this basic information, we know little else about oarfish, despite the species being cited frequently as a possible origin of sea serpent and mermaid legends [1, 5]. In particular, our understanding of oarfish life history is lacking. A few descriptions exist of the early *Regalecus* developmental stages [6, 7], but these are replete with errors from insufficient sampling, misidentification or uncertain identification, and failure to preserve voucher specimens [1]. The only reliable record of the early stages of *Regalecus* is a report of eggs from the western Pacific, identified using DNA barcoding techniques [8], and a juvenile (13.7 mm in standard length) identified from developed morphological features [9].

In January 2019, we obtained a recently dead pair of mature oarfish and successfully performed artificial insemination with their gametes, producing fertilized eggs. These were then hatched into live oarfish larvae, observable for the first time. This is the first reliable record of fertilized eggs developing into early larval fish in the Lampridiformes.

## Materials and methods

### Adult fishes

**Fig. 1:**
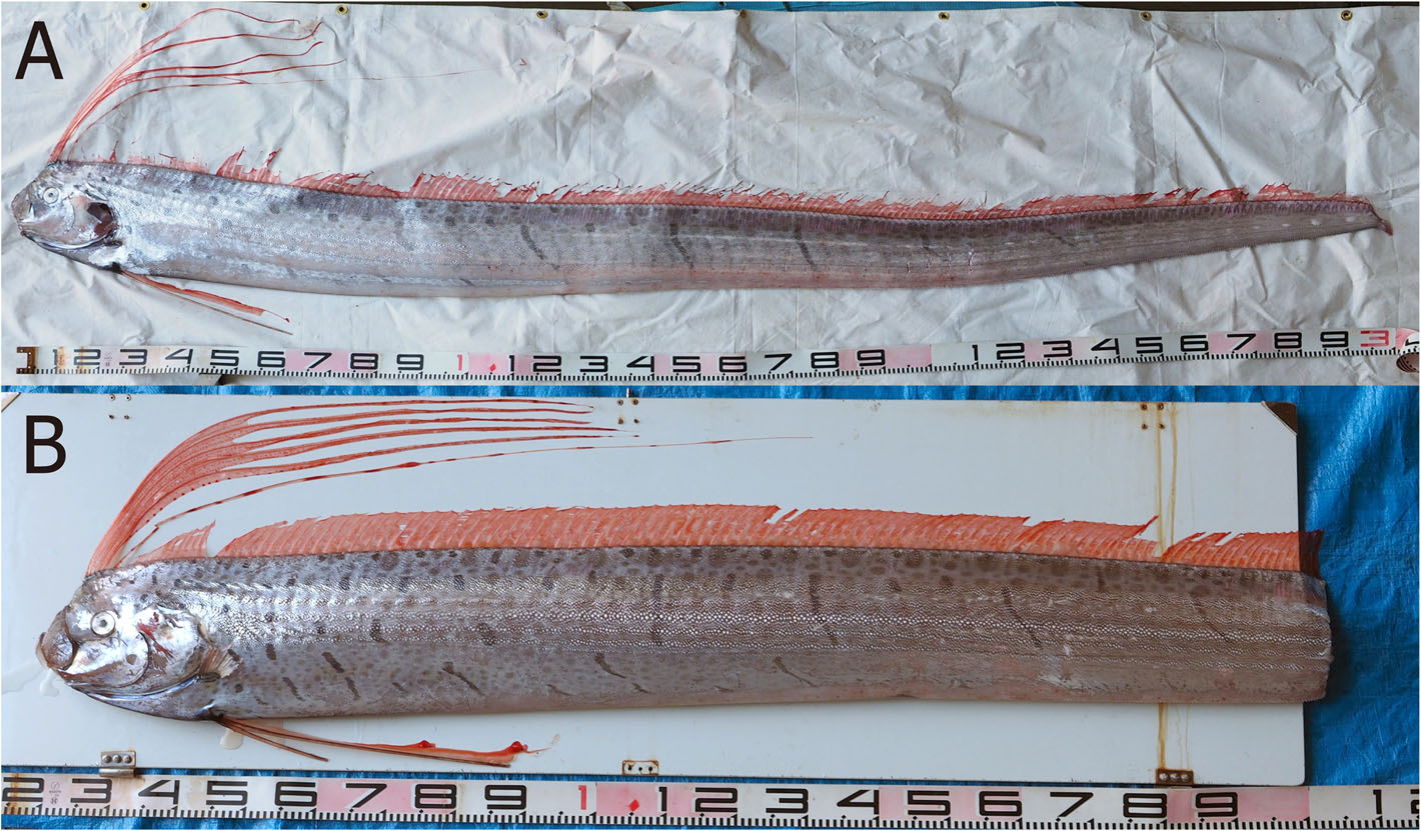
*Regalecus russelii* pair used for artificial insemination. **a**: female, **b**: male

During early morning of January 29, 2019, two living *Regalecus russelii* (Fig. 1) were captured in a set net (c.a. 360 m × 60 m) for commercial fishes on the coast of Okinawa-jima Island (26° 22′ 48″ N, 127° 42′ 45″ E; 40 m depth). They were transferred to a car equipped with a seawater tank. By that point, one fish had already died; the other was alive, but had lost its tail during capture. Within an hour of being conveyed to the Okinawa Churaumi Aquarium, the remaining specimen also died. These individuals were identified as *R. russelii* from the number of gill rakers (male: 52, female: 56) and pre-anal dorsal fin rays (male: 67, female: 70) following a previous study [1]. The two individuals measured 1220 mm in pre-anal length. They were discarded because there were no tanks of adequate size to preserve these two large specimens. For future genetic identification, the muscle tissue of both fish were deposited in the collection of the Okinawa Churashima Foundation (OCF) as OCF-P4061 and OCF-P4062.

### Artificial insemination and egg incubation

The female possessed one pair of ovaries; upon dissection, a large amount of transparent eggs spilled from the end of the oviduct. The other individual was a male with a pair of testes containing white liquid, which was confirmed under microscopic observation to be sperm with high motility. Artificial insemination was performed using the dry method, mixing eggs and sperm in a 4 L container before adding seawater. After treatment, non-floating eggs were removed and the remainder incubated at 20 °C. The fertilized eggs (about 400) were maintained with weak aeration in a 30 L tank from 1 day after fertilization (DAF) to 7 DAF. Subsequently, approximately 100 eggs were transferred to a 100 L tank to reduce mortality risk. Water temperatures were kept at 20.5–22.5 °C. Observations of fertilized eggs throughout development were performed under a microscope from 1 to 3 DAF and then daily after 7 DAF until hatching.

### Larvae care and treatment

The 19 newly hatched larvae were separated into three tanks: nine and seven larvae were kept in two 30 L tanks, while the remaining three were kept in a 100 L tank. The water temperature was 22 °C. Rotifers (*Brachionus plicatilis*) and brine shrimp (*Artemia nauplii*), enriched with highly unsaturated fatty acids, were provided as food for the larvae. The larval behavior was filmed daily using a compact digital camera (OLYMPUS TG-5). Total video length was 29 min 23 s; swimming, body posture, and other characteristic behaviors were visible. Analysis used a video (19 min and 35 s long) taken 0–2 days after hatching (DAH). The videos from 3 and 4 DAH were excluded because the larvae appeared unable to swim and did not exhibit any characteristic behaviors.

Except for one larva that died on the day of hatching, those that appeared close to dying or had died were taken as specimens for morphological observations. All living specimens were euthanized via anesthesia overdose before fixing. Specimens were preserved in 5% formalin and deposited in the OCF collection. Total length (TL), notochord length (NL), pre-anal length (PAL), head length (HL), and eye diameter (ED) were measured under a microscope (KEYENCE VHX-1000), from five specimens that did not flex when fixed.

The larval specimens (OCF-P04196) were cleared and double-stained for bone and cartilage following published protocol [10]. Photographs of clear and double-stained specimens were obtained using the microscope’s filming function.

## Results

### Development of fertilized eggs

**Fig. 2:**
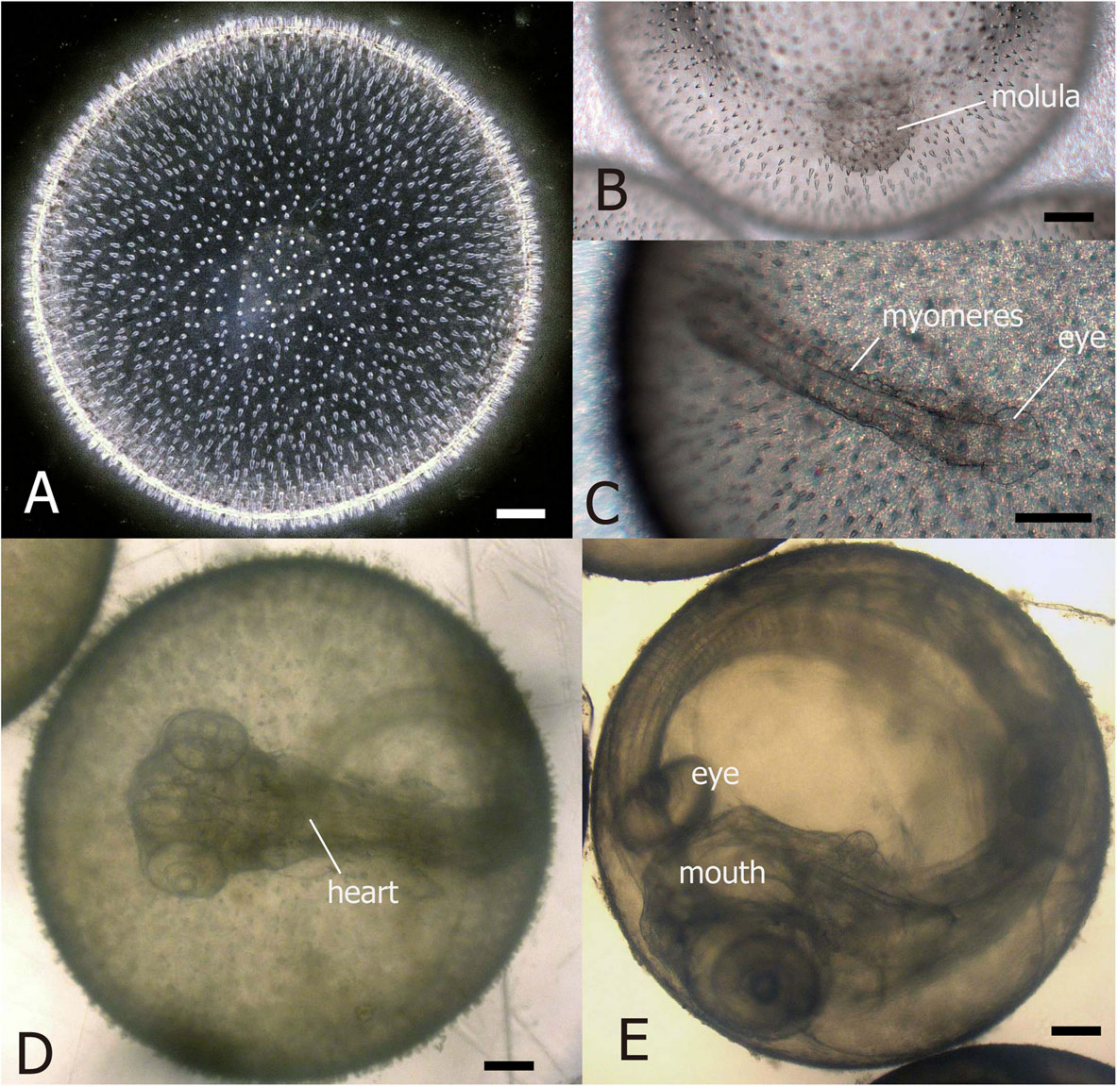
Fertilized eggs of *Regalecus russelii.***a**: immediately after fertilization, **b**: morula at 1 day after fertilization (DAF), **c**: embryo at 7 DAF, showing eye and myomere development, **d**: egg at 11 DAF, E: egg just before hatching (16 DAF). Scale bars: 0.2 mm

The eggs were spherical and 2.07–2.20 mm in diameter (*n* = 38), with homogeneous yolk, no oil globules, and a surface covered with numerous conical short spines (Fig. 2a). About 400 floating eggs had developed into a morula at 1 DAF (Fig. 2b). At 7 DAF, 300 eggs remained, while the head, eyes, notochord, and myomeres developed (Fig. 2c). The tip of the tail was released from the yolk at 9 DAF, and the heart beats were confirmed with the body development at 11 DAF, when 270 eggs remained (Fig. 2d). This number declined to 40 by 15 DAF, when embryo mouth opened and the small surface spines became inconspicuous. At 16 DAF, pigmentation appeared in the eyes and the dorsal fin ray elongated (Fig. 2e). The first individual hatched at 17 DAF, while 18 larvae hatched at 18 DAF; the remaining eggs failed to hatch.

### Larval morphology

**Fig. 3:**
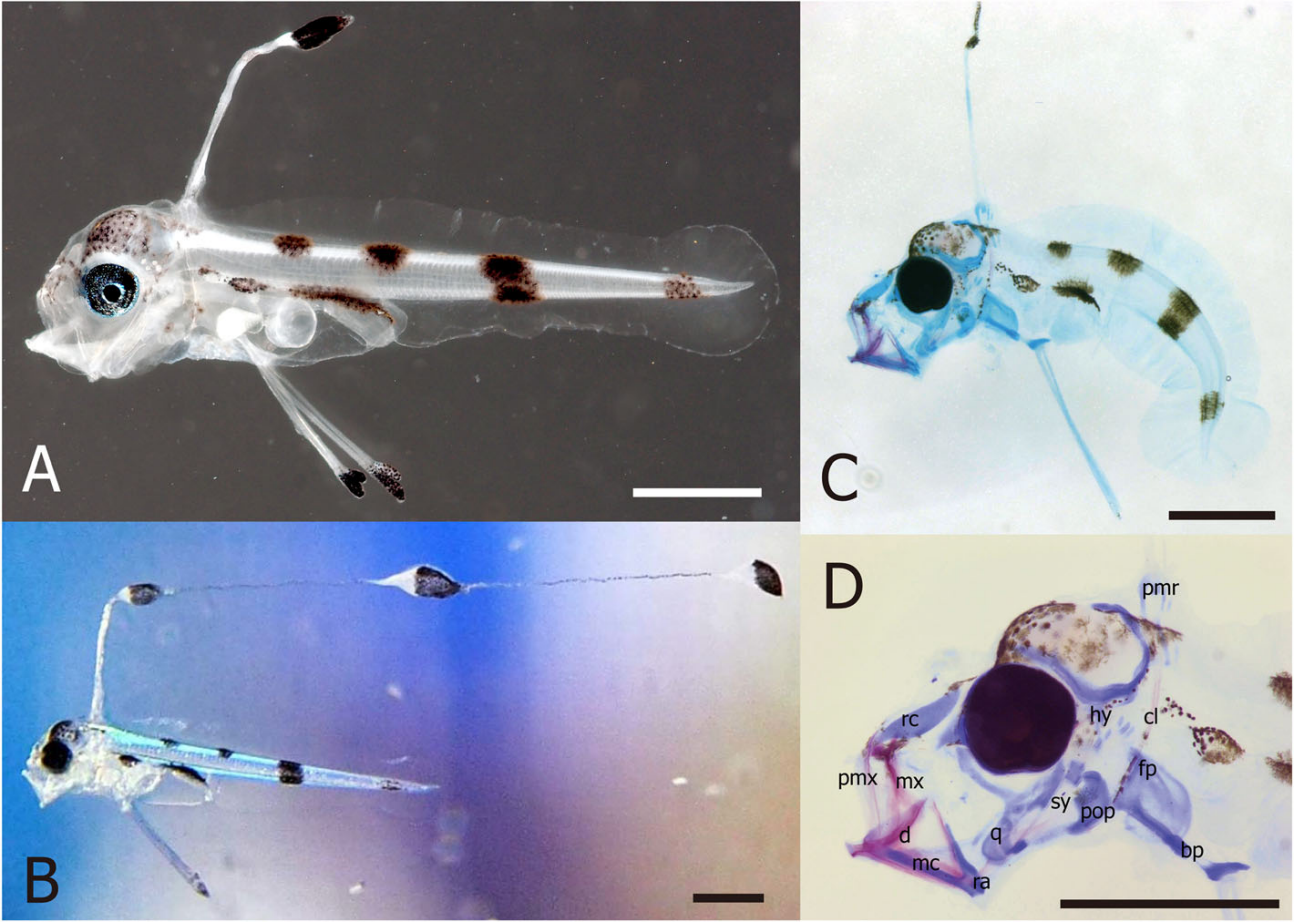
Larvae of *Regalecus russelii*. **a**: larval specimen (5.5 mm notochord length (NL); 2 days after hatching (DAH); OCF-P04188, dorsal elongated filament was lost), **b**: living larvae ca. 6 mm NL (1 DAH), **c**: transparent specimen (5.5 mm NL; 4 DAH; OCF-P04196), **d**: close-up of transparent specimen head. Anatomical abbreviations: bp, basipterygium; cl, cleithrum; d, dentary; fp, fin plate cartilage; hy, hyomandibular; mc, Meckelian cartilage; mx, maxilla; pmr, proximal middle radial; pmx, premaxilla; pop, preopercle; q, quadrate; ra, retroarticular; rc, rostal cartilage; sy, symplectic. Scale bars: 1.0 mm

Larval measurements are shown in Table 1, and morphological characters are shown in Fig. 3a and b. Newly hatched larvae (5.5–6.3 mm NL) had moderately elongated and laterally compressed bodies (Table 1). The proportion of first dorsal and pelvic fin rays in the notochord length exceeded by 0.31 ± 0.05 and 0.23 ± 0.01 times (mean ± SD, *n* = 5), respectively. With the exception of pectoral fins, other fin rays were not developed. The single dorsal fin ray had elongated into a filament (averaging 1.8 times the notochord length, measured from videos, *n* = 6) with three large bulges exhibiting granule-like pigmentation (Fig. 3). The proportions of PAL/NL, HL/NL, and ED/HL were 0.46 ± 0.02, 0.19 ± 0.01, and 0.40 ± 0.03 (means ± SD), respectively (Table 1). None of the specimens had any head spination (Fig. 3). The myomere count was 27 in the pre-anal area and 101–107 in the tail; however, the myomeres in the tail tip were unclear. The yolk sac was completely absorbed by 3 DAH. Melanophore pigmentation was scattered on head and snout; dorsally located over air bladder and hindgut; in two patches on dorsal trunk, one dark bar on mid-tail, one faded bar on tail tip; present on tip of pelvic fin, and on swellings of the elongated dorsal fin ray.

**Table 1:** Measurements of larvae obtained from the first artificial insemination of *Regalecus russelii*

Specimen No.	DAH	TL (mm)	NL (mm)	PAL (mm)	HL (mm)	ED (mm)
OCF-P04185	0	6.40	6.10	2.90	1.12	0.46
OCF-P04186	1	6.50	6.30	2.90	1.18	0.45
OCF-P04188	2	5.70	5.50	2.55	1.16	0.43
OCF-P04191	3	6.10	5.95	2.70	1.08	0.48
OCF-P04192	4	5.80	5.53	2.35	1.03	0.43

### Osteological development

Observations of the transparent larval specimens showed no bones or related vertebrae from the posterior part of the trunk (Fig. 3c, d). However, the bones around the head were well developed while connective tissue in jaws developed as cartilage, with ossification occurring only in premaxilla, maxilla, dentary, and part of the symplectic bones. The pectoral fin, elongated dorsal fin, and pelvic fin were also developed. The cleithrum was ossified, while other elements (pectoral fin plate, proximal middle radial, and basipterigium) were cartilage.

### Larval survival and behavior

A total of 19 larvae hatched, but the number of living individuals decreased to 17 at 1 DAF, 12 at 2 DAH, 8 at 3 DAH, and 0 by 4 DAH.

Video analysis (Movie S1) showed that larvae swam mainly using the pectoral fin, although they sometimes moved quickly using their tails when bait plankton touched their bodies. Larvae often (56.3% of the time) maintained a downward-facing body posture, while spending 4.7% of the time oriented slightly upward and 4.2% upside down. They were horizontally oriented and moved laterally 13.6% of the time. Larvae quickly rotated multiple times to escape entanglement if their elongated dorsal fin wrapped around their bodies. They also slowly rotated when changing direction. Such rotational behavior occurred 19.8% of the time. Although we observed larvae opening their mouth six times, it was unclear whether this represented attempts to eat prey plankton, and we did not notice direct feeding behavior.


**Additional file 1 Movie S1**. Recording of oarfish larval behavior


## Discussion

Eggs hatched 18 DAF, in line with a previous study indicating that lampridiform fishes hatch after 10–20 days [11]. Ours is the first study to track lampridiform eggs from fertilization to hatching. Our observations revealed that oarfish eggs develop much more slowly than eggs of other pelagic teleostean fishes. The eggs we describe here shared similar morphological characteristics to those of oarfish eggs obtained from the ocean and identified through DNA barcoding [8]. Although we caught the two mature oarfish in winter, a previous study identified oarfish eggs around the Marshall Islands in summer [8]. Thus, the spawning season of this species may be relatively long (summer–winter), although the exact duration remains unclear. Our capture of the matured pair in a set net, along with the discovery of stranded individuals oarfish with matured gonads [12], support the hypothesis that this species rises to the surface for spawning. Currently, over 40 records of stranded oarfish were found between the temperate and tropical regions of Japan [4]. Future studies should examine gonads from these specimens to further clarify oarfish reproductive biology.

Oarfish larvae had similar morphological features as other lampridiform larvae (*Lophotus lacepede*, *Trachipterus* spp., and *Zu cristatus*) [13, 14]. However, previous reports of lampridiform larvae are unreliable, as they contain the multiple errors [1]. The present study provides the first reliable description of lampridiform larvae, specifically from *R. russelii*. The bodies of newly hatched oarfish larvae are more compressed than those of adults, but both larvae and adults possess elongated dorsal and pelvic fins. The smallest juvenile ever captured in the ocean (13.7 mm NL) similarly had an adult-like elongated body and developed dorsal fin rays on the anterior half of the body [9]. This similarity between hatched larvae and juveniles suggests that the larval body elongates soon after hatching.

Larval and adult swimming behaviors were completely different. Adults swim using their dorsal fins in a head-up position [15], whereas larvae often maintained a downward position and swam using their pectoral fins. We do not know the reason for this larval posture, nor do we know the function of their remarkably elongated dorsal-fin filament and its ornaments; we did not observe any situations in which larvae used the filament. Furthermore, the filament often tangled around the body and appeared to impede movement. We note, however, that these behaviors occurred in captive conditions and may differ from behaviors in the wild.

Our osteological observations revealed a lack of ossification in connective tissue at the posterior part of the head, including vertebrae. However, upper and lower jaws were well-ossified, and tissue related to opening the mouth were developed as cartilage. We also observed ossification of the cleithrum, which supports the pectoral fin. These results are consistent with the observation that larvae frequently opened their mouths and swam using their pectoral fins. The biggest mystery in our study was that the larvae ate nothing, despite possessing mouths with apparently sufficient feeding function. This observation suggests that, unlike other teleosts, oarfish do not feed on active plankton at the sea surface. Nevertheless, we observed frequent “yawning” behavior that may be related to a very specific feeding ecology, although we do not know what type of food the larvae consume. In addition, however, we also cannot exclude the possibility that the captive environmental conditions caused this feeding dysfunction.

## Conclusions

This is the first report of successful artificial insemination in oarfish and provides the first reliable record of development from fertilized eggs to early larval stages in Lampridiformes. Eggs and sperm from a recently dead pair of matured fishes were used for artificial insemination. The eggs developed more slowly than other teleostean pelagic eggs and required 18 days for hatching. Hatched larvae developed head composition and elongated dorsal and pelvic fin rays. These morphological features, including the pigmentation pattern, were similar to characteristics reported for other lampridiform larvae in previous studies.

Striking larval behaviors included maintenance of a downward-facing position and swimming using pectoral fins. We were unable to determine the function of the remarkably elongated dorsal-fin filament and its ornaments. Larvae did not feed, and all larvae died within 4 DAH in spite of possessing a well-developed mouth. These findings contribute significantly to our understanding of early oarfish life history, although the captive conditions of this study may differ greatly from natural environmental conditions. In addition, the fact that the mature male and female oarfish were caught with a coastal set net suggests that this species may migrate to the surface for spawning.

## Data Availability

The datasets used and analyzed during the current study are available from the corresponding author on reasonable request.
